# Cathodal Transcranial Direct Current Stimulation Over the Right Temporoparietal Junction Suppresses Its Functional Connectivity and Reduces Contralateral Spatial and Temporal Perception

**DOI:** 10.3389/fnins.2021.629331

**Published:** 2021-02-17

**Authors:** Guo Dalong, Li Jiyuan, Zhou Yubin, Qin Yufei, Yang Jinghua, Wang Cong, Jia Hongbo

**Affiliations:** ^1^Air Force Medical Center, Air Force Medical University, Beijing, China; ^2^Beijing Shijitan Hospital, Beijing, China; ^3^Department of Basic, Air Force Engineering University, Xi’an, China

**Keywords:** internal model, temporoparietal junction, transcranial direct current stimulation, vestibular nerve, self-motion, spatial perception, functional connectivity

## Abstract

The temporoparietal junction plays key roles in vestibular function, motor-sensory ability, and attitude stability. Conventional approaches to studying the temporoparietal junction have drawbacks, and previous studies have focused on self-motion rather than on vestibular spatial perception. Using transcranial direct current stimulation, we explored the temporoparietal junction’s effects on vestibular-guided orientation for self-motion and vestibular spatial perception. Twenty participants underwent position, motion, and time tasks, as well as functional magnetic resonance imaging scans. In the position task, cathodal transcranial direct current stimulation yielded a significantly lower response in the −6, −7, −8, −9, −10, −11, and −12 stimulus conditions for leftward rotations (*P* < 0.05). In the time task, the temporal bias for real transcranial direct current stimulation significantly differed from that for sham stimulation (*P* < 0.01). Functional magnetic resonance imaging showed that cathodal transcranial direct current stimulation suppressed functional connectivity between the temporoparietal junction, right insular cortex, and right supplementary motor area. Moreover, the change in connectivity between the right temporoparietal junction seed and the right insular cortex was positively correlated with temporal bias under stimulation. The above mentioned results show that cathodal transcranial direct current stimulation induces immediate and extended vestibular effects, which could suppress the functional connectivity of the temporoparietal junction and in turn reduce contralateral spatial and temporal perception. The consistent variation in temporal and spatial bias suggested that the temporoparietal junction may be the cortical temporal integrator for the internal model. Moreover, transcranial direct current stimulation could modulate the integration process and may thus have potential clinical applications in vestibular disorders caused by temporoparietal junction dysfunction.

## Introduction

The temporoparietal junction (TPJ) is a pivotal multimodal region, located at the intersection of the lateral occipital cortex, inferior parietal lobule, and posterior end of the superior temporal sulcus (Mars et al., 2012). Although the detailed functional characteristics and anatomical boundaries of the TPJ remain contested, this region is considered to be a critical nerve center that receives upstream information from visual, auditory, somatosensory, thalamic, and limbic brain regions; all this information converges on the TPJ, where it is integrated and processed (Decety and Lamm, 2007). Conventional investigations on the role of the TPJ have investigated whether patients with TPJ lesions can perform specific tasks attributable to the TPJ. Based on lesion studies, the TPJ is associated with vestibular function, motor-sensory ability, attitude stability, perception-action coupling, and other egocentric or allocentric spatial orientation abilities (Ro et al., 1998; Perennou et al., 2000; Ventredominey et al., 2003; Kaski et al., 2016). However, using traditional methods requires a large enough sample of patients affected only by TPJ lesions, which may be unrealistic since brain damage normally diffuses to adjacent regions and thus hampers the generalizability of conclusions. Moreover, the human brain has a powerful ability to compensate for functional loss (i.e., via plasticity) after neurocortical injury; thus, conclusions drawn from patients with cerebral injuries may be inaccurate.

Non-invasive physical stimulation methods, such as transcranial direct current stimulation (tDCS) and transcranial magnetic stimulation (TMS), have been used to explore brain function and circumvent such drawbacks of previous studies. *Via* tDCS, a low and direct electrical current is applied to the scalp or one electrode is placed on the scalp and another on the arm or shoulder to temporarily elevate or suppress the function of specific brain regions (Clark et al., 2012; Coffman et al., 2014). By transiently down-regulating the arousal level of the TPJ through cathode electrode stimulation, the TPJ is specifically hyperpolarized and its firing rate suppressed (Koganemaru et al., 2017). Thus, tDCS can mimic the symptoms of minor TPJ lesions and help delineate TPJ’s function (Stewart et al., 2001).

Some studies have used tDCS to probe TPJ’s physiological function (Arshad et al., 2014; Mai et al., 2016). Arshad et al. employed cathodal tDCS to inhibit the left TPJ, which disrupted the parietal inter-hemispheric balance and resulted in asymmetrical vestibular-ocular reflex (VOR) suppression; they also found that the right hemisphere plays a dominant role in vestibular cortical processing (Arshad et al., 2014). Kyriakareli et al. (2013) applied electrodes over the bilateral TPJ and found that tDCS increases the VOR and perceptual thresholds, regardless of the polarity of the electrodes. Another study also found that the use of cathodal tDCS inhibits cortical excitability in the right TPJ, thus showing the significant value of this technology for clinical treatment and rehabilitation (Mai et al., 2016). Although these studies with tDCS have found that the TPJ plays a crucial role in vestibular-ocular and perception thresholds, they have focused on the perception of self-motion (“am I moving?”) and neglected vestibular spatial perception (“where am I?”), which also plays a key role in spatial orientation. Furthermore, the exact mechanisms by which the TPJ exerts its function in vestibular-guided orientation remain unknown.

Here, using tDCS and functional magnetic resonance imaging (fMRI), we performed a double-blinded, sham-controlled study to explore how the right TPJ affects vestibular-guided orientation, not only for self-motion perception but also for vestibular spatial perception. Furthermore, we hypothesized that cathodal tDCS could suppress TPJ’s functional connectivity (FC), and that the TPJ could constitute the cortical temporal integrator in vestibular-guided orientation.

## Materials and Methods

### Participants

Twenty right-handed participants (all male; mean age 20.6 ± 1.7 years) without any tDCS contraindications (cardiovascular disease, stroke, or mental illnesses) or disorders that would impair vestibular-guided orientation (paraequilibrium, otolithiasis, or Meniere’s disease) were enrolled. Written voluntary consent for participation was obtained after detailed information about the potential risks of the test and precautions was provided. The study was approved by the local Ethics Committee of the Air Force Medical Center according to the Code of Ethics of the World Medical Association (Declaration of Helsinki).

### TDCS Paradigm

**FIGURE 1:**
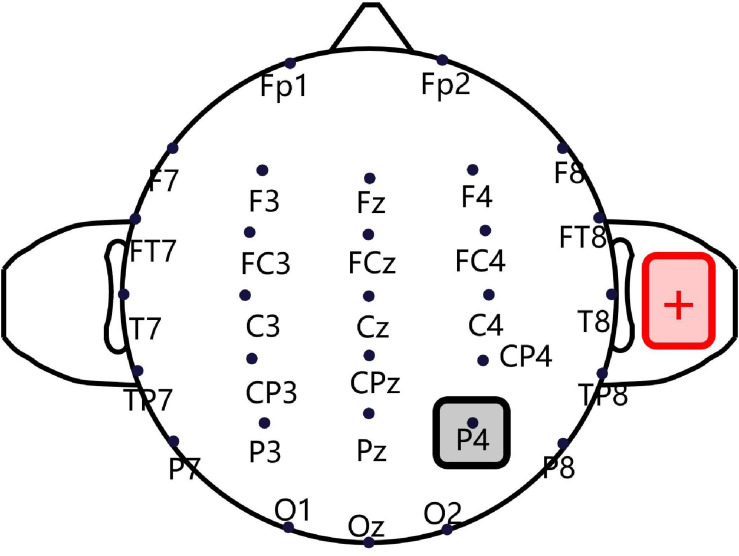
Transcranial direct current stimulation montage. Saline-soaked cathodal sponge electrodes (5 cm × 5 cm) were applied over the right P4 region, according to the International 10/20 electroencephalogram (EEG) system, and a larger anodal electrode (5 cm × 7 cm) was placed on the right shoulder deltoideus triangularis.

A CE-approved constant-current, battery-driven stimulator (NeuroConn, Ilmenau, Germany) was used for tDCS. As shown in Figure 1, a saline-soaked cathodal sponge electrode (5 cm × 5 cm) was applied over the right P4 region, according to the International 10/20 electroencephalogram system, using medical bandages. A larger anodal electrode (5 cm × 7 cm) was similarly placed on the right shoulder’s deltoideus triangularis in such a way as to avoid skin burns and reduce irritation (Mckinley et al., 2013; Mcintire et al., 2014). A standard 64-channel EEG recording cap was used to locate the cathodal electrode (Turi et al., 2015; Moliadze et al., 2018). A constant current was ramped up to 2 mA in 10 s and sustained for 15 min, and the maximum current density never exceeded 0.08 mA/cm^2^, as per current safety limits (Bikson et al., 2009). For the sham condition, an identical montage was used, except that the current was only sustained for 30 s, after which current intensity was reduced to zero, to cause an itching sensation but without incurring any cortical stimulation. Previous tDCS fMRI studies have shown that this sham stimulation only causes a slight effect on the cerebellum without any significant influence on brain connectivity (Park et al., 2013).

### Position Task

**FIGURE 2:**
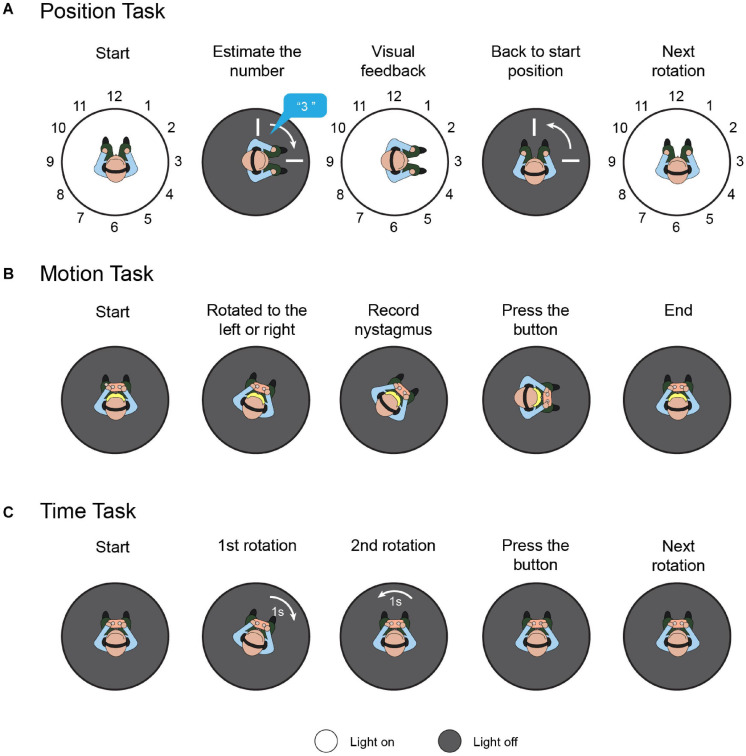
Task protocols. **(A)** Position task. Participants were instructed to sit on a rotational vestibular chair; the inner face of the chair enclosure was marked with 12 numbers. First, the participant faced number 12. Then, the participant was randomly rotated to the left or right. The participants were asked to estimate the number they were facing without providing them with any visual cues. The light was then switched on to reveal that number and the participants returned to the initial position. This process was repeated to involve all orientation directions, velocities, and angle amplitudes. **(B)** Motion task. Participants sat holding a control panel with two buttons. The chair rotated to the left or right randomly from rest with the acceleration increasing by 0.5 *deg*/s^2^ every 3 s, and rotation-induced vestibular nystagmus was recorded. When the participants felt they were rotating, they pressed the corresponding button. **(C)** Time task. Each time task comprised two rotations with equal duration and opposite direction, in random order. After the two rotations, the participant was instructed to press the button corresponding to the rotation with the longer duration.

A position task was used to assess spatial orientation based solely on vestibular cues (Figure 2A). Participants were instructed to sit on a rotational vestibular chair (system 2000, Interacoustics, Denmark). The inner face of the chair enclosure was marked with 12 numbers (1 to 12) at equal intervals (30°), like a clock face. First, the participant faced number 12, while the laboratory was maintained in total darkness. White noise was played via a headset to hide auditory cues. Then, the participant rotated to the left or right through an angle of 30°×*N*(*N* = 1, 2…12) in a randomized and ergodic order, with raised peak cosine angular velocities of 80°/s, 100°/s, and 120°/s. After every rotation, participants were asked to estimate the number they were facing without having any visual cues. The light was then switched on to reveal that number. Then, the participant returned to the initial position, and the light was switched off. The above process was repeated 72 times in total, to involve all orientation directions, velocities, and angle amplitudes. In healthy individuals, without stimulation, the regression slope for the rightward rotation should be equal to the leftward rotation (Donaldson et al., 2015). To quantify the directivity of the spatial orientation ability, “position bias” can be calculated as the rightward rotation regression slope versus the leftward rotation regression slope.

### Motion Task

Recent studies have demonstrated that VOR and vestibulo-perceptual procedures can be altered separately, both clinically (Seemungal et al., 2011) and experimentally (Panichi et al., 2011), which indicates that the cerebral cortex may influence the respective functions differently. To evaluate the participants’ motion perception comprehensively, both the vestibular-ocular threshold and vestibulo-perceptual threshold were measured in the dark (Figure 2B). Participants sat in the rotational vestibular chair, holding a control panel with two buttons (left and right). The chair rotated to the left or right randomly from rest, with the acceleration increasing by 0.5 *deg*/s^2^ every 3 s. When the participants felt they were rotating left or right, they pressed the corresponding button, and the vestibulo-perceptual thresholds were recorded. Rotation-induced vestibular nystagmus was recorded by a head-mounted, infrared, binocular video oculography system (CHARTR VNG, ICS Medical, Taastrup, Denmark), and the threshold for the VOR was calculated based on the nystagmus. These were considered the vestibular-ocular thresholds (Seemungal et al., 2007). Throughout the test, the participants were secured in the chair by a safety belt, were in the dark, and wore a headset with white noise to eliminate visual and auditory interference. A more detailed experimental protocol and the used data-processing algorithm are described elsewhere (Seemungal et al., 2007; Cutfield et al., 2011).

### Time Task

Since the vestibular cues were integrated by the time required to derive the perceived position, we performed a task to evaluate the participants’ motion-time perception ability. Similar to the former tasks, the participants were stabilized in the rotational vestibular chair by a safety belt; white noise was played over the headset, and the laboratory was kept in total darkness during the test (Figure 2C). Each time task comprised two rotations with equal duration and opposite direction, in random order. After the leftward and rightward rotations, the participant was instructed to press the button corresponding to the rotation with the longer duration. For each participant, seven consecutive time tasks were randomly assigned with different durations, i.e., 1, 1.5, 1.75, 2, 2.5, 3, and 4 s. The rotation amplitude was limited to 180°, and the maximum rotation speed was set to 60°/s. To quantify tDCS-induced time perception directivity, temporal bias was defined as the proportion of participants pressing the right button versus the total task number. Theoretically, for healthy individuals, the average temporal bias should be 0.5, which means that they have no orientation time-tendency.

### FMRI Protocol and Image Analysis

Resting state fMRI (rs-fMRI) was performed at the People’s Liberation Army Hospital no. 309 (Beijing, China), employing a SIEMENS Magnetom Trio Tim 3.0T equipped with a 12-channel head matrix coil. In total, two scans were performed for each participant, immediately after they underwent real and sham tDCS in the scanner suite.

At the initial phase of the first scan, an MPRAGE sequence was used to acquire a high-resolution T1-weighted anatomical image [flip angle = 150°, echo time (TE) = 9 ms, repetition time (TR) = 2000 ms, voxel size = 1 mm × 1 mm × 1 mm, field of view (FOV)= 320 mm × 320 mm, 196 slices]. Two functional scans adopted the same protocol, including 210 functional images, applying a standard echo-planar imaging sequence (flip angle = 90°, TE = 30 ms, TR = 2 s, voxel size = 3 mm × 3 mm × 4 mm, FOV = 256 mm × 256 mm, matrix size = 64 × 64, 45 slices, gap = 1 mm). During scans, participants were instructed to remain relaxed, breathe smoothly, and keep staring at a fixation cross.

The scanned rs-fMRI data were preprocessed by SPM12^1^ using Matlab (The MathWorks Inc., Natick, MA, United States) in the following sequence: discarding the first five volumes, time correction, realignment, regression, filtering (0.01–0.08 Hz), normalization, and smoothing. Then, the CONN toolbox was adopted to perform seed-to-whole-brain FC analyses, and the right TPJ (MNI: 57, 29, 21), centered at the superior temporal gyrus including the supramarginal gyrus, was selected as the seed for exploring its role in vestibular-guided orientation (Ionta et al., 2014). In the first FC analysis level, time series correlation analysis was performed between the seed region’s time series and all brain voxels to produce seed-to-whole-brain Fisher transformation maps. Afterward, group-level analyses were performed utilizing these maps in CONN. To calculate the main effects, repeated measures analysis of variance was adopted with sham tDCS and real tDCS. The contrast was set to sham tDCS > real tDCS, and the procedure was controlled by age. The cluster level threshold was *P* < 0.05, false discovery rate-corrected, with more than 100 voxels per cluster.

To examine if changes in FC after cathodal stimulation were related to temporal bias under the real tDCS condition, we ran a linear regression analysis of each participant’s FC difference (sham – real) with temporal bias under the real tDCS condition as the regressor of interest, with the statistical significance threshold set at *P* < 0.05, Bonferroni-corrected for multiple comparisons.

### Procedures

**FIGURE 3:**
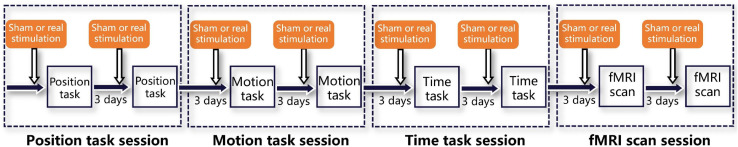
Experimental procedures. The whole experiment consisted of four sessions, i.e., a position task session, motion task session, time task session, and fMRI scan session. Each session was separated from the previous one by 3 days to eliminate aftereffects and included two tasks or scans, also separated by 3 days. Immediately before both tasks or scans, sham and real stimulation were delivered, in random order.

As shown in Figure 3, each participant underwent four sessions, i.e., a position task session, motion task session, time task session, and fMRI scan session. Each session contained two tasks or scans, and every task or scan maintained a 3-day time lag to avoid tDCS- or rotation-induced aftereffects. Before each task, a sham or real stimulation was randomly executed for each participant. In addition, both the participants and the laboratory assistants who operated the rotatory chair, provided instructions, and executed the stimulation protocols were blinded to the stimulation order, which guaranteed double-blinding of the study. Notably, all tasks or scans were sustained for less than 30 min to avoid efficacy attenuation induced by cathodal stimulation (Mcintire et al., 2014).

The task results were plotted to display the vestibular-guided orientation ability using Origin (version OriginPro 2019; Origin Lab, Northampton, MA, United States). A two-tailed, paired *t*-test was used to compare responses between conditions. Statistical analyses were performed using SPSS 20, and differences were considered significant for *P* < 0.05.

## Results

The performance of all participants conformed to the regulations mentioned above, and none of the participants reported symptoms of skin burns or persistent vertigo.

### Position Task

**FIGURE 4:**
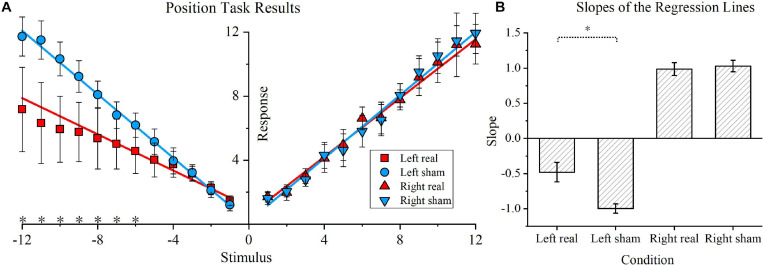
Position task and position bias results. **(A)** Position task results. The blue circle and inverted blue triangle represent the sham transcranial direct current stimulation (tDCS) condition for the leftward and rightward rotations, respectively. Correspondingly, for the real tDCS condition, a red square and red triangle represent the responses of the two stimulus directions. The linear regression lines for the real and sham tDCS condition are also denoted by blue and red lines, respectively. Asterisks are used to indicate significant differences between the real and sham tDCS conditions in the specific stimulus position. **(B)** Slopes of the regression lines for the four conditions. The slopes of the regression lines for the left real, left sham, right real, and right sham condition were calculated for each participant. The mean values and standard deviations of the four slopes are shown, and the vertical lines represent standard errors of the mean.

The position task results and respective regression lines of real and sham conditions are shown in Figure 4A, while regression line slopes for four conditions (left real, left sham, right real, and right sham) are shown in Figure 4B. As shown in Figure 4A, for leftward rotations, the real tDCS condition showed significantly lower responses for the −6, −7, −8, −9, −10, −11, and −12 stimulus conditions (*P* < 0.05 for all conditions). In contrast, for rightward rotations, no significant difference was found in any stimulus point. Furthermore, the linear equation solved by the least squares fitting method was *y* = −0.47*x* +   1.54 for the leftward rotation stimulated by real tDCS, and the corresponding equation for the left sham condition was *y* = −1.00*x* +  0.14. Similarly, the fitting linear equations for the right real and right sham conditions were *y* = −0.95*x* +  0.38 and *y* = 1.01*x* +  0.02, respectively. The slopes of the regression lines for the left real, left sham, right real, and right sham conditions were calculated for each participant and are shown in Figure 4B. For leftward rotation, the absolute slope values were significantly lower in the real tDCS condition (−0.47 ± 0.14) than in the sham tDCS condition (−0.99 ± 0.07) (*P* < 0.01). For rightward rotations, no significant difference was found between the real (0.98 ± 0.09) and sham condition (1.03 ± 0.08). Furthermore, the mean position bias value for the real tDCS condition (−0.49 ± 0.16) significantly differed from that of the sham tDCS condition (−0.97 ± 0.09) (*P* < 0.01).

### Motion Task

**FIGURE 5:**
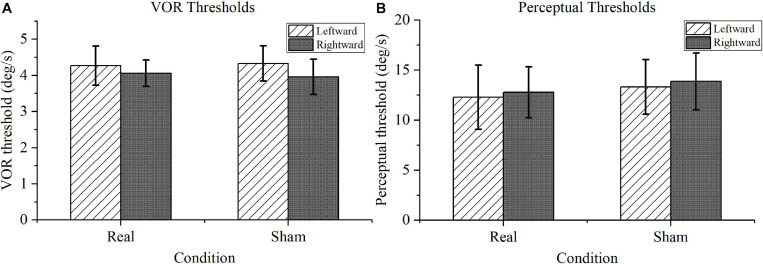
Motion task results. **(A)** Vestibular-ocular reflex (VOR) thresholds. There was no significance difference in performance under the real transcranial direct current stimulation (tDCS) and sham conditions for VOR thresholds in leftward or rightward rotation. **(B)** Motion perception thresholds. Compared with the perceptual thresholds under sham conditions, the motion perception thresholds under real tDCS showed no marked difference in either direction.

For the VOR threshold results shown in Figure 5A, no significant difference was found between the real and sham conditions, either for the left or right rotation (*P* > 0.1). Furthermore, for the perceptual threshold shown in Figure 5B, no marked differences were identified between the real and sham conditions (*P* > 0.1). Notably, the perceptual thresholds were within the normal range for the real tDCS condition according to the previous literature (Cousins et al., 2013).

### Time Task

**FIGURE 6:**
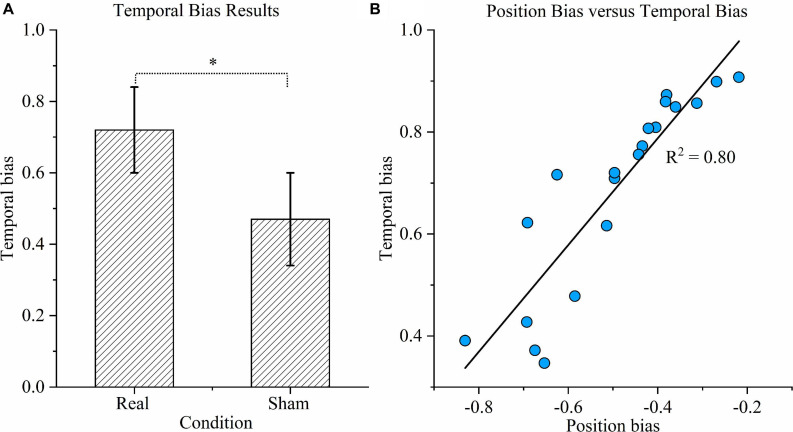
Time task results. **(A)** Temporal bias results. Temporal bias was defined as the proportion of participants indicating that the duration of the right rotation was longer when the rotations in opposite directions were in fact equal. Asterisks indicate significant differences between real and sham transcranial direct current stimulation (tDCS) conditions (*P* < 0.01). **(B)** Correlation between position bias and temporal bias. Correlation between position bias in position task performance and temporal bias in time task performance across all participants under the real tDCS condition. The coefficient of determination denoted *R*^2^ in the figure is the proportion of temporal bias that can be predicted from position bias.

As shown in Figure 6A, the temporal bias under sham stimulation (0.47 ± 0.13) was similar to the theoretical value (0.5) and significantly different from the value under the real condition (0.72 ± 0.12) (*P* < 0.01). Figure 6B exhibits the relationship between position bias and temporal bias in the real condition. The coefficient of determination was 0.80, indicating that 80% of the variation in temporal bias could be explained by the position bias; hence, the two biases were highly correlated.

### FC Changes With Sham and Real Stimulation

We measured changes in FC between the sham and real tDCS (Table 1). After cathodal tDCS, FC decreased between the right TPJ seed and the right insular cortex (IC), right supplementary motor area (SMA), right temporal occipital fusiform cortex, and right thalamus.

**TABLE 1:** Main effects of cathodal tDCS on FC (sham tDCS > real tDCS).

**Region (label)**	**MNI coordinates**	**T-statistics**	**Cluster size**	**Cluster *p*-value**
Right insular cortex (IC r)	−38 −6 +2	5.13	263	<0.001
Right supplementary motor area (rSMA)	+15 −7 +62	5.16	206	<0.01
Right temporal occipital fusiform cortex (TOFusC r)	+37 −54 −25	5.64	163	<0.01
Right thalamus	+25 −26 −9	5.21	101	<0.01

*The main effects of cathodal tDCS on FC that apply to the contrast of sham tDCS > real tDCS. The *x*, *y*, and *z* coordinates refer to the MNI coordinate system; t-statistics and cluster *p*-values correspond to the peak voxels within the anatomical region(s) specified in the left column. For all contrasts, increased activity is reported at a cluster level threshold of *p* < 0.05 (false discovery rate corrected). FC, functional connectivity; MNI, Montreal Neurological Institute; tDCS, transcranial direct current stimulation.*

**FIGURE 7:**
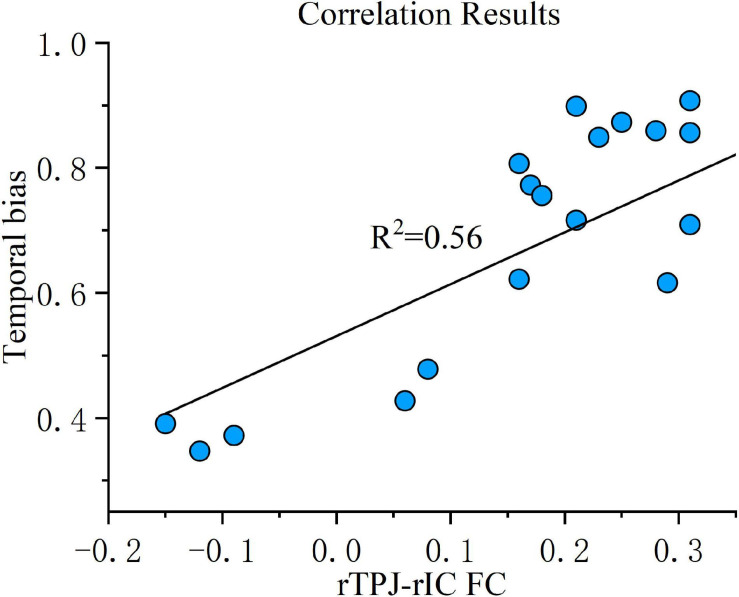
Correlation results. FC change between the right TPJ and the right IC was correlated with temporal bias across all subjects under the real tDCS condition. The coefficient of determination, denoted *R*^2^ in the figure, is the proportion of temporal bias that can be predicted from FC variation. FC, functional connectivity; rTPJ, right temporoparietal junction; rIC, right insular cortex.

Correlation analysis was then conducted to evaluate the relationship between the FC changes (sham-real) and temporal bias under the real tDCS condition. The change in connectivity between the right TPJ seed and the right IC was positively correlated with the change in temporal bias under the real condition (*p* < 0.001) (Figure 7). On the other hand, the decrease in connectivity between the right TPJ seed and the right SMA, right temporal occipital fusiform cortex, and right thalamus were not significantly correlated with temporal bias after real tDCS.

### Exclusion of Galvanic Vestibular Stimulation

To exclude the possibility that our results were caused by galvanic vestibular stimulation (GVS), which occurs when transmastoid direct current modulates peripheral vestibular activity (Cutfield et al., 2011; Kyriakareli et al., 2013), we measured torsional nystagmus (the mean feature associated with GVS). No torsional nystagmus was found in the videos recorded during the real tDCS condition, indicating that tDCS did not extend to peripheral vestibular afferents.

## Discussion

In the present study, we aimed to explore the role of the TPJ in vestibular-guided orientation, not only for self-motion perception but also for vestibular spatial perception, using tDCS and fMRI.

The position task revealed that the right cathodal tDCS suppresses responses in seven left rotations (Figure 4) but does not result in any significant difference for the right rotations. No regression line slope for leftward rotation was higher than any for rightward rotation in the real tDCS condition, which suggests that there was no opposite trend. Furthermore, the position bias results indicated that cathodal tDCS above the right TPJ alters the direction tendency of the position estimation. In the time task, cathodal tDCS caused a higher temporal bias compared to that in the sham condition (Figure 6A), implying that cathodal tDCS reduces the duration estimation for the leftward rotation.

The motion task demonstrated that the right cathodal tDCS has no significant impact on VOR thresholds and perceptual thresholds (Figure 5). In contrast, a previous study showed that tDCS applied over the TPJ increases both VOR thresholds and vestibular system-mediated movement perception (Kyriakareli et al., 2013). Electrode montage discrepancy may be the key reason for this disparity. Kyriakareli et al. (2013) performed bilateral stimulation using an anode and a cathode symmetrically placed over the right and left TPJ, respectively. Furthermore, they supposed that bilateral tDCS could import background “noise” and thus generally perturb the neuronal activity of the TPJ. However, our results contradict this hypothesis, because unilateral tDCS can also introduce a background noise that would not alter movement perception. A more convincing hypothesis is that the VOR and motion perception could be bilaterally encoded in the brain’s cerebral cortex and require concurrent left and right stimulation to exert an appreciable impact. This hypothesis is also supported by a neuroanatomical study in ballet dancers (Yuliya et al., 2015) and is congruent with the results of a caloric labyrinthine test after unilateral tDCS (Arshad et al., 2014).

Regarding the rs-fMRI results, we applied seed-based connectivity analysis to study the FC of the right TPJ using a pre-set contrast: sham tDCS > real tDCS. The results indicated that cathodal tDCS suppresses connectivity of the right TPJ, right IC, and right SMA. According to a previous study, the IC is both a motor association and a vestibular area anatomically connected to the TPJ (Augustine, 1996). Moreover, the IC was shown to be specifically implicated in the integration of concordant sensory signals and self-perception—crucial for spatial perception (Farrer and Frith, 2002; Vogeley et al., 2004; Devue et al., 2007; Tsakiris et al., 2007). On the basis of their co-activation during vestibular stimulation, the coupling between the right TPJ and right IC has been considered pivotal for spatial orientation (Bottini et al., 1994). The present FC-fMRI results also revealed that the temporal bias under the real tDCS condition is positively correlated with the changes of FC between the right TPJ and the right IC, which may suggest that reducing FC between the right TPJ and the right IC could in turn damage temporal perception. The SMA is also involved in spatial navigation, visuo-spatial processing, and in accumulating and integrating spatial information (Boly et al., 2007; Leek et al., 2016; Cona et al., 2017), all necessary in the process of forming spatial orientation perception. The present rs-fMRI results further corroborate former findings by revealing that the network facilitating spatial orientation comprises the right TPJ, right IC, and right SMA, and that suppressing their coupling could impair contralateral spatial perception.

The surprisingly consistent fluctuation in spatial and temporal biases was particularly noteworthy. One possibility is that cathodal tDCS may enhance specific cognitive abilities, which could affect temporal and spatial performance simultaneously, e.g., an attention mechanism. Numerous studies have demonstrated that tDCS leads to attention improvement and facilitates mission completion (Gladwin et al., 2012; Mcintire et al., 2014; Nelson et al., 2014). However, these studies all stimulated the dorsolateral prefrontal cortex region by anodal tDCS, and no previous reports have investigated whether cathodal tDCS deployed above the TPJ region improves or reduces attention. Furthermore, contrary to the theory that the TPJ may intervene in both temporal and spatial information in vestibular-guided orientation through attention, our rs-fMRI results revealed that the spatial perception-related network, rather than the attention-related network, was deactivated upon cathodal tDCS of the TPJ. Another possibility in view of the congruent deficits in both temporal and position bias is that there may also be a relationship between the neural mechanisms underlying spatial orientation and motion duration perception. The concept of “mathematical integration” could be the underlying logic maintaining the spatial orientation and motion duration perception in a synchronous fluctuation. It implies that the brain derives the distance estimation by continuously sampling self-motion velocities and adding it over time. Therefore, the TPJ may encode vestibular-guided movement in a form that preserves the relationship between traveled distance (s), motion velocity (v), and motion duration (t) i.e., *s* = ∫*v*.*dt* The above hypothesis is also supported by a recent brain lesion study (Kaski et al., 2016). Moreover, Merfeld et al., have proposed a mathematical model, termed the “observer model,” to explain how the brain integrates sensory information in an effort to mimic spatial orientation perceptual responses (Clark et al., 2019). Many studies have reported on specific cortical and subcortical brain regions that can construct an internal representation of perceived current motion and orientation in space, showing that there is an internal model in the brain, with some brain regions corresponding to its specific computing elements (Laurens and Angelaki, 2017; Clark et al., 2019). In the observer model, the vestibular system receives acceleration information that needs to be integrated twice to obtain estimates of position and velocity (Newman, 2009). Green et al. (2005) showed that brainstem and cerebellar neurons could be the first integrators of this internal model. The TPJ could be the second integrator, by continuously receiving self-motion velocity signals and accumulating this velocity signal over time to derive position, similar to the widely accepted brainstem integrator for eye movement control (Arshian et al., 2014). In agreement with the internal model theory, Indovina et al. (2005) used fMRI to demonstrate that the vestibular network, including the TPJ, is engaged in orientation and prediction when the acceleration on the screen is coherent with natural gravity. Moreover, Bosco et al. (2008) used TMS to disturb TPJ function and found that this only affects the participants’ interception of motion, consistent with acceleration under gravity, suggesting that the TPJ might specifically dispose visual-motion information and timing signals according to an internal model under gravitational law constraints. Moreover, considering that the IC is believed to be involved in the encoding of time intervals and time judgment (Kosillo and Smith, 2010; Wittmann et al., 2010), our correlation results, by showing that the FC between the IC and the TPJ is positively associated with temporal bias, may suggest that the IC continuously encodes time duration perception, and sends this message to the TPJ, which is used to integrate velocity signals over time to derive position. Although this hypothesis could not be definitely demonstrated, it is the most consistent with our experimental results.

However, several limitations of this study should be emphasized here. First, the abovementioned results, which lay the basis for our hypotheses, only provide unilateral behavioral and imaging evidence; therefore, left tDCS and bilateral tDCS studies (left anodal right cathodal or left cathodal right anodal) are warranted for further verification. In addition, although all participants experienced a 3-day washout period after the first task, this process may not completely rule out sustained psychological and physiological after-effects caused by repeated tDCS and tasks, which could conceivably have affected the task or scan results. Even though all tasks or scans were sustained for less than 30 min to avoid efficacy attenuation induced by cathodal stimulation, we cannot establish that the efficacy of tDCS could be maintained unchanged when the participants tackled the tasks or underwent the scans. Moreover, owing to the low spatial specificity of tDCS, we cannot completely rule out the possibility that our results could also derive by the activation or suppression of other cortical regions. Therefore, a TMS study for TPJ is still needed to confirm the specificity of our results. Considering the above limitations, the results of this study should be interpreted carefully. More rigorous studies with larger sample sizes will be needed to validate the inferences and conclusions presented here.

## Conclusion

The results of this study showed that cathodal tDCS induces immediate and extended vestibular effects, which suppress the FC of TPJ with the right IC and right SMA, and in turn reduce the contralateral spatial and temporal perception. Our data demonstrated that the TPJ region plays a key role in spatial and temporal perception in vestibular-guided orientation, and that dominant TPJ suppression cannot alter motion perception. The consistent variations in temporal and spatial bias and the positive correlation between FC change (between the right TPJ and the right IC) and temporal bias under the real tDCS condition suggest that the TPJ may represent the cortical temporal integrator for the internal model, continuously receiving velocity information which, when integrated over time, yields position estimations. Because tDCS of the TPJ could modulate the above integration process, it may have potential clinical applications in vestibular disorders caused by TPJ dysfunction.

## Data Availability Statement

The original contributions presented in the study are included in the article/supplementary material, further inquiries can be directed to the corresponding authors.

## Ethics Statement

The studies involving human participants were reviewed and approved by the Air Force Medical Center. The patients/participants provided their written informed consent to participate in this study.

## Author Contributions

GD and JH: conception and study design. GD, LJ, and ZY: data collection or acquisition. ZY, WC, and QY: statistical analysis. GD, JH, and ZY: interpretation of results. GD, YJ, QY, ZY, WC, and JH: drafting the manuscript or revising it critically for important intellectual content. All authors approved the final version to be published and agreement to be accountable for the integrity and accuracy of all aspects of the work.

## Conflict of Interest

The authors declare that the research was conducted in the absence of any commercial or financial relationships that could be construed as a potential conflict of interest.

## Footnotes

1 http://www.fil.ion.ucl.ac.uk/spm/software/spm12/
